# 2504: Defining peripheral B cell tolerance in pemphigus vulgaris

**DOI:** 10.1017/cts.2017.51

**Published:** 2018-05-10

**Authors:** Nina Ran, Christoph Ellebrecht, Eun Jung Choi, Aimee Payne

**Affiliations:** School of Medicine, University of Pennsylvania, Philadelphia, PA, USA

## Abstract

OBJECTIVES/SPECIFIC AIMS: Pemphigus vulgaris (PV) is a potentially fatal blistering disease caused by autoantibodies to the keratinocyte adhesion protein desmoglein 3. Several other autoimmune diseases have defective B cell tolerance checkpoints, resulting in the accumulation of self-reactive and polyreactive B cells. METHODS/STUDY POPULATION: The present work aims to determine whether PV patients develop normal tolerance to self-antigens other than desmoglein 3, as a potential “first hit” in the development of autoimmunity. We use FACS to isolate single B cells at 4 developmental stages from 8 PV patients. We perform single-cell RT-PCR to amplify each B cell receptor, produce monoclonal antibodies, and screen these for autoreactivity using ELISA/IF to several self-antigens. At each B cell stage, we compare the frequencies of self-reactive and polyreactive B cells to those found in healthy controls. RESULTS/ANTICIPATED RESULTS: We anticipate similar frequencies between PV patients and controls, suggesting that the B cell repertoire in PV patients develops normally at early checkpoints. DISCUSSION/SIGNIFICANCE OF IMPACT: The absence of generalized reactivity would distinguish PV from other autoimmune diseases and would show that PV arises from a specific break in tolerance to a single self-antigen (desmoglein 3) during late B cell maturation. Such a result would further support PV as an ideal candidate for targeted immunotherapy.

